# Supplementary figures and images for: Fecal Metabolic Profiling of Breast Cancer Patients during Neoadjuvant Chemotherapy Reveals Potential Biomarkers
Source: Molecules. 2021 Apr 14;26(8):2266. doi: 10.3390/molecules26082266 (PMC8070723; doi:10.3390/molecules26082266)

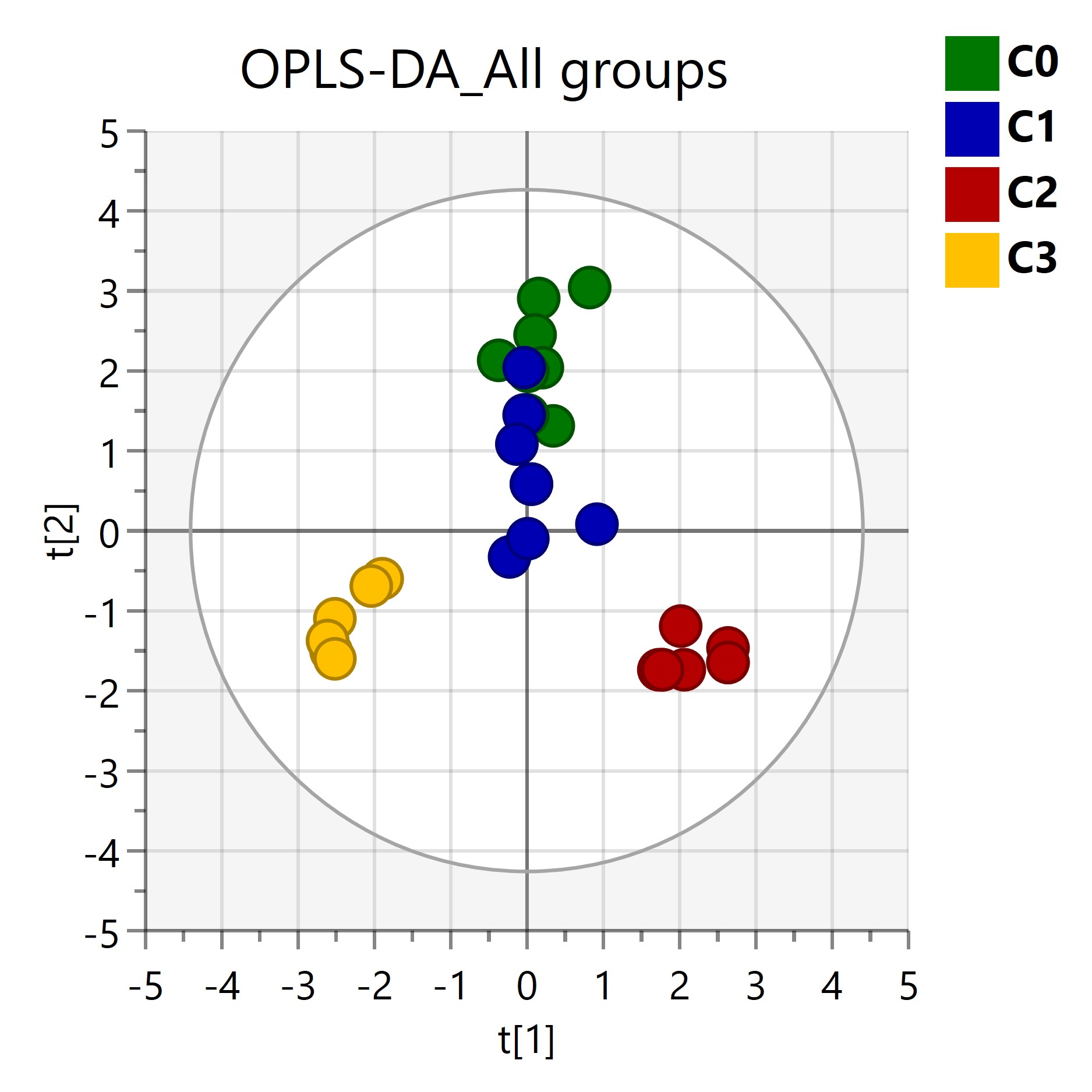

Supplement: Supplementary file 1 [file molecules-26-02266-s001.zip › Supplementary figure S1.jpg]
